# RNAi screens for Rho GTPase regulators of cell shape and YAP/TAZ localisation in triple negative breast cancer

**DOI:** 10.1038/sdata.2017.18

**Published:** 2017-03-01

**Authors:** Patricia Pascual-Vargas, Samuel Cooper, Julia Sero, Vicky Bousgouni, Mar Arias-Garcia, Chris Bakal

**Affiliations:** 1 Dynamical Cell Systems Team, Cancer Biology, Institute of Cancer Research, 237 Fulham Road, London SW3 6JB, UK; 2 Department of Computational Systems Medicine, Imperial College London, South Kensington Campus, London SW7, UK

**Keywords:** RHO signalling, Breast cancer, RNAi, High-throughput screening, Cellular imaging

## Abstract

In order to metastasise, triple negative breast cancer (TNBC) must make dynamic changes in cell shape. The shape of all eukaryotic cells is regulated by Rho Guanine Nucleotide Exchange Factors (RhoGEFs), which activate Rho-family GTPases in response to mechanical and informational cues. In contrast, Rho GTPase-activating proteins (RhoGAPs) inhibit Rho GTPases. However, which RhoGEFs and RhoGAPS couple TNBC cell shape to changes in their environment is very poorly understood. Moreover, whether the activity of particular RhoGEFs and RhoGAPs become dysregulated as cells evolve the ability to metastasise is not clear. Towards the ultimate goal of identifying RhoGEFs and RhoGAPs that are essential for TNBC metastasis, we performed an RNAi screen to isolate RhoGEFs and RhoGAPs that contribute to the morphogenesis of the highly metastatic TNBC cell line LM2, and its less-metastatic parental cell line MDA-MB-231. For ~6 million cells from each cell line, we measured 127 different features following the depletion of 142 genes. Using a linear classifier scheme we also describe the morphological heterogeneity of each gene-depleted population.

## Background & Summary

The spread of metastatic cells around the body, and subsequent colonisation of distant sites, is the leading cause of breast cancer patient death^1,2^. In order to metastasise, cancer cells dynamically change shape^3–5^. In all eukaryotic cells, cell shape is regulated by Rho GTPases, which are inactive in a GDP-bound form, but undergo a conformational change upon the binding of GTP, which allows them to activate and recruit downstream effectors^6^. Rho GTP Exchange Factors (RhoGEFs) activate Rho GTPases by inducing the exchange of bound GDP for GTP on Rho GTPases^7^; whereas Rho GTPase-activating proteins (RhoGAPs) inhibit Rho GTPase activity by accelerating GTP hydrolysis^8–10^.

Triple negative breast cancers (TNBC) lack expression of estrogen-, progesterone- and human epidermal growth factor type 2 (HER-2) receptors^11^. These characteristics make TNBC difficult to treat as they do not respond to endocrine treatments or targeted therapies^11,12^. Given the critical role RhoGEFs and RhoGAPs play in regulating cell shape, and their presumptive role in metastasis, RhoGEFs and RhoGAPs are attractive targets for therapeutic intervention in TNBC^9,10^. However, both RhoGEFs and RhoGAPs are large families of signalling enzymes (82 and 67 members respectively^6^), and the subset of these that couple TNBC cell shape to their environment has not been defined.

Towards the ultimate goal of gaining insight into which RhoGEFs and RhoGAPs are essential for the shape changes that underpin TNBC metastasis, we performed RNAi screens of all human RhoGEFs and RhoGAPs to identify genes that contribute to the morphogenesis of the highly metastatic LM2 TNBC cell line^13^. To determine if the activity of particular RhoGEFs and RhoGAPs become dysregulated during the evolution of metastasis, we also performed the screens in their less-metastatic parental cell line MDA-MB-231; based on the intuition that RhoGEFs and RhoGAPs essential for LM2, but not MDA-MB-231, morphogenesis are likely candidates for genes whose activity becomes dysregulated during the metastatic process. Two Dharmacon libraries were used per cell line to minimise off-target effects: siGENOME and ONTARGET*Plus*. The ONTARGET*Plus* library contains siRNAs with a patented dual strand modification to limit off-target effects, thus having less off-target effects than the siGENOME library. Libraries were arrayed over 384-well plates, and each screen was performed in duplicate (8 plates per cell line). Briefly, cells were reverse transfected on day 1, fixed on day 3, stained on days 3 and 4, and imaged from day 4 onwards. Cells were stained for α-tubulin, polymerized F-actin, and the transcriptional co-activators YAP and TAZ^14,15^. The α-tubulin and actin stains give information on how each RhoGEF and RhoGAP affects the cytoskeleton, as well as being excellent labels for cytoplasm segmentation that facilitates quantification of overall shape. YAP and TAZ are two transcriptional co-activators that are regulated by the Hippo signalling pathway^14,15^. They translocate to the nucleus upon their activation by mechanical cues—in particular cell contractility and spreading^16^. The antibody used in these assays recognises both YAP and TAZ proteins. Nuclear translocation of YAP/TAZ serves as a proxy for activation of transcriptional activity^17^. Thus, by simultaneously quantifying the translocation of YAP/TAZ with cell shape, we can determine if gene depletion has affected cell shape in a manner that has resulted in the activation of YAP/TAZ mechanotransduction (i.e., increased spreading or contractility), or whether the cell shape change has occurred through alternate means (i.e., decreased viability); which provides some mechanistic insight into how gene depletion may be affecting shape. This data will be useful for elucidating a group of RhoGEFs and RhoGAPs which contribute to the cell shape of TNBC cells and could drive TNBC metastasis. The identification of these genes would warrant their further study in 3D cultures, and finally *in vivo*.

**Figure 1:**
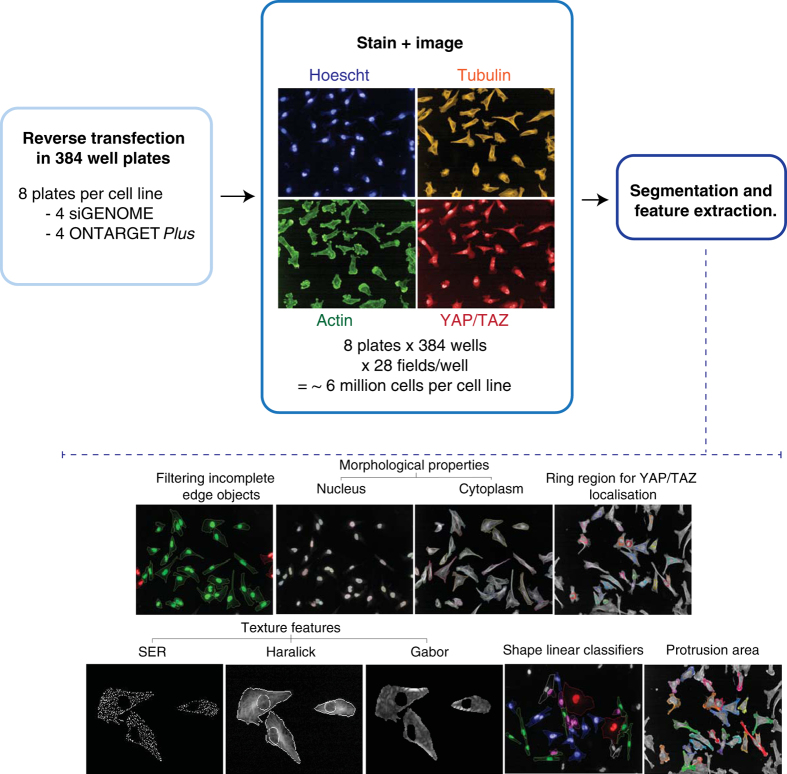
Experimental workflow. This study consisted of RNAi screens to isolate RhoGEFs and RhoGAPs that are essential for the morphogenesis of the highly metastatic TNBC cell line LM2, and its less-metastatic parental cell line MDA-MB-231. The screening approach is depicted in a sequential manner starting with the reverse transfection, followed by staining and imaging, and ending on image analysis.

We present both image data, as well as 127 features describing the average cell shape and YAP/TAZ localisation for each well (siRNA) (Fig. 1). As a means of performing feature reduction and accounting for the heterogeneity of populations^18^, we implemented a method to identify the proportion of cells in each population that can be classified as belonging to one of five classes with a particular shape (Data records 1–4). This method is similar to those we have previously used to perform feature reduction^18–21^, and conceptually similar to methods pioneered in classic morphological screens^22^.

Both images and numerical data are suitable for re-analysis by different image analysis packages and statistical/computational techniques respectively.

## Methods

### Cell culture

LM2s (subpopulation 4,172 from MDA-MB-231) were obtained from Joan Massagué^13^ and stably express a triple-fusion protein reporter construct encoding herpes simplex virus thymidine kinase 1, green fluorescent protein (GFP) and firefly luciferase^13^. MDA-MB-231 cells (obtained from Janine Erler) and LM2 cells were grown in Dulbecco’s Modified Eagle Medium (DMEM) supplemented with 10% heat-inactivated fetal bovine serum (FBS) and 1% penicillin/streptomycin in T75 falcons. Both cell lines were grown at 37 °C and supplemented with 5% CO_2_ in humidified incubators.

### High throughput RNA interference screening

#### Overview of RNAi screening methodology

Two human RhoGEF and RhoGAP libraries were used: siGENOME and ONTARGET*Plus* (Dharmacon). Both libraries were made up of SMARTpools, where each gene is targeted by a pool of 4 different siRNA strands per siRNA. Each siRNA library was arrayed across 2 black, optically clear bottom, 384 well Cell Carrier plates (PerkinElmer). Individual siRNAs were arrayed in duplicate per plate, and each plate was screened in duplicate, resulting in 4 plates per screen per cell line: 1A, 1B, 2A and 2B. As positive controls *LATS1* (Dharmacon cat # M-004632-00) and *YAP* (Dharmacon cat # L-012200-00) siRNA were introduced on columns 23 and 24 with a minimum of 4 wells per control per screen. *LATS1* siRNA was used as a control for our ability to assess YAP/TAZ localisation, as in the absence of LATS1 YAP/TAZ nuclear localisation is expected to increase^14^. *YAP* siRNA was used as a control to assess our ability to monitor both YAP levels and localisation. As a control to assess our ability to transfect cells we used *ECT2* siRNA (Dharmacon cat # L-006450-00, # M-006450-00) which results in multinucleate cells^23,24^.

YAP/TAZ nuclear translocation can be sensitive to cell density, and thus some siRNAs might affect YAP/TAZ localisation simply because they reduce cell numbers^17^. To account for density dependent effects cells were plated at increasing densities on columns 1, 2, 23 and 24. By performing a linear regression, or similar analysis on these samples users can identify the relationship between YAP/TAZ localisation and cell density. However, in these screens presented here no siRNAs had significant effects on cell number.

Mock transfected cells served as our negative controls, due to our previous observation that non-targeting siRNAs result in phenotypic changes (unpublished data).

All liquid dispensing steps were carried out using a Multidrop Dispenser (Thermo Scientific), except for siRNA plating on the assay plates which was carried out using the acoustic liquid handler Echo 550 (Labcyte Inc).

#### Screening reagents

- Dharmacon siGENOME siRNA library.
- Dharmacon ONTARGET*Plus* siRNA library.
- Opti-MEM Reduced Serum Media (Thermo Fisher, Gibco Life Technologies, cat # 31985062).
- Lipofectamine ® RNAimax reagent (Invitrogen, cat # 13778150).
- 8% methanol free formaldehyde in PBS (16% pre-made solution Thermo Scientific, cat # 28908), final concentration 4%.
- PBS containing 0.05% of the preservative Sodium Azide (Sigma, cat # S2002-25G) (all PBS referred to in this study).
- Triton-X-100 (Sigma, cat # T9284), 10% in PBS.
- Bovine Serum Albumin (BSA) (Sigma, cat # A2153-50G) 2% in PBS, filtered.
- Triton-X-100, 0.01% and 0.5% BSA in PBS solution for antibody incubations.
- Mouse YAP/TAZ antibody (Santa Cruz Biotechnology, cat # sc-101199) 1 μgml^−1^ (1:1,000).
- Rat α-tubulin antibody (Bio-Rad, cat # MCA77G) 1 μgml^−1^ (1:1,000).
- AlexaFluor 647 goat anti-mouse (Life Technologies, cat # A21235) 2 μgml^−1^ (1:1,000).
- AlexaFluor 568 goat anti-rat 1:1,000 (Life Technologies, cat # A11077) 2 μgml^−1^ (1:1,000).
- Phalloidin 488 (Invitrogen, cat # A12379) 1:1,000 from 300 U in 1.5 ml stock.
- 2′-(4-Hydroxyphenyl)-5-(4-methyl-1-piperazinyl)-2,5′-bi(1H-benzimidazole) trihydrochloride (Hoechst) (Sigma Aldrich, cat # 33258) 10 μgml^−1^ (1:1,000).

#### Day 0: Plating siRNAs

40 nl (0.08 pmol) siRNA from the siGENOME and ONTARGET*Plus* libraries (stock concentration of 20 μM) were arrayed using the acoustic liquid handler Echo 550 prior to transfection and kept at −80 °C.

#### Day 1: Reverse transfection

Pre-stamped siRNA plates were thawed at room temperature for 30 to 60 min prior to use. Subsequently, 5 μl of Opti-MEM® Reduced Serum Media were added per well. 5 min later, 5 μl of mix containing Opti-MEM and RNAimax reagent in a 125:1 ratio were added, and plates were spun at 1,000 r.p.m. for 1 minute. Plates were incubated at room temperature for 20 min, so as to allow siRNA-RNAimax complexes to form. Cells were then seeded at 1,000 cells per well (30,000 cellsml^−1^) in 30 μl DMEM (10% FBS, 1% penicillin/streptomyocin), resulting in a total volume of 40 μl per well: 40 nl siRNA (final concentration of 20 nM), 5 μl Opti-MEM, 5 μl of Opti-MEM and RNAimax mix (125:1) plus 30 μl of cells.

#### Day 3: Immunofluorescence staining

Automated handling of plates on the Cell::Explorer robot station was carried out using the PlateWorks software (Perkin Elmer). Cells were fixed 48 h post transfection by addition of 40 μl of warm 8% formaldehyde in PBS (final concentration 4%) for 15 min. Cells were washed with PBS three times, permeabilised with 0.1% Triton-X-100 in PBS for 10 min and blocked with 2% BSA diluted in PBS for 2 h. All antibody incubations were performed in solution containing 0.5% BSA, 0.01% Triton-X-100 dissolved in PBS in a 1:1,000 ratio. Plates were washed three times with PBS between steps using a Microplate Washer (BioTek). Antibodies were added sequentially to avoid any possible bleedthrough, with the mouse primary antibody for both YAP and TAZ (final concentration 1 μgml^−1^), being added first and left overnight.

#### Day 4: Immunofluorescence staining and imaging

Staining procedures were continued in the following order with each antibody left on for 1.5 h: AlexaFluor 647 goat anti-mouse (final concentration 2 μgml^−1^) to detect YAP/TAZ, rat primary anti α-tubulin antibody (final concentration 1 μgml^−1^), AlexaFluor 568 goat anti-rat to detect α-tubulin (final concentration 2 μgml^−1^) and Phalloidin 488 combined in the same step. Finally the nuclear stain Hoechst (Sigma Aldrich, cat # 33,258, final concentration 10 μgml^−1^) was added for 15 min, upon which it was removed and 15 μl of PBS were dispensed. All cells were imaged using an automated Opera Quadruple Enhanced High Sensitivity (QEHS) spinning-disk confocal microscope (PerkinElmer) with 20x air lens. Lasers and their corresponding filters used were: 405(450/50), 561 (600/40), 488 (540/751), 640 (690/50).

### Quantitative real-time PCR (qRTPCR)

Reverse-transfections with ONTARGET*Plus* SMARTpool *DOCK5*, *ECT2, ARHGEF7* (Dharmacon cat # L-018931-00-0005, # L-006450-00-0005, and # L-009616-00-0005 respectively); and deconvoluted *ARHGEF7* (Dharmacon cat # LU-009616-00-0025) siRNAs, were performed in the same manner as for the RhoGEF/GAP screens. LM2 and MDA-MB-231 cells were plated at a density of 50,000 cellsml^−1^ in 6 well plates. RNA was extracted using the phenol:chloroform (TRIzol®, ThermoFisher cat # 15596026), and RNAeasy kit (Qiagen cat # 74,104) according to the manufacturer’s protocol. RNA was converted to cDNA using a cDNA conversion kit (Applied Biosystems cat # 4387406) according to the manufacturers protocol. qRTPCR was performed on a QuantStudio® Flex Real-Time PCR system, using PCR mastermix SyBR green (Applied Biosystems cat # 4309155) and the following primers: DOCK5-forward GCTTTGAACTTCAGCTCTGG, DOCK5-reverse CCACATGTCCCGGATTCTAA; ECT2-forward CCCGAGCTGGAGAAACTATG, ECT2-reverse ACCCTATGGAAAGGGAATGC; ARHGEF7-forward GATGCCACACGATGAAAACCC, ARHGEF7-reverse GTGTCTCTCGAGCTCTTTGA. Fold changes were calculated on quadruplicate technical replicates using double delta Ct analysis and normalised to GAPDH.

### Data collection and analysis

#### Image processing and segmentation

Images were processed and analysed using the Columbus 2.6.0. Software Platform (PerkinElmer). To identify single cells, nuclei were segmented using the Hoechst signal. The cytoplasm was subsequently segmented based on the identified nuclei utilising α-tubulin intensity. Cells touching the image border were filtered out.

#### Feature extraction

In total 126 features describing intensity, morphological, and texture properties were extracted for each cell. Additionally the fraction of neighbouring cells was calculated. All features are given as the average feature value across all single cells for a given well of the screen. These features could be broadly grouped into different types:

1. Nucleus area, roundness, width, length, width to length ratio.
2. Cell area, roundness, width, length, width to length ratio.
3. Cytoplasm area, roundness, width, length, width to length ratio.
4. Membrane region (protrusions) area.
5. YAP/TAZ stain intensity for nucleus, ring region, whole cell, cytoplasm, nucleus region.
6. Ratio of nuclear region YAP/TAZ intensity to ring region YAP/TAZ intensity.
7. Log_10_ of ratio of nuclear region YAP/TAZ intensity to ring region YAP/TAZ intensity (YAP/TAZ ratio)
8. Ratio of eroded nuclear region YAP/TAZ intensity to ring region YAP/TAZ intensity.
9. Log_10_ of ratio of eroded nuclear region YAP/TAZ intensity to ring region YAP/TAZ intensity.
10. Ratio of nuclear region intensity to nuclear area.
11. Ratio of nuclear region YAP/TAZ to nuclear area.
12. Ratio of cell area to whole cell YAP/TAZ.
13. Ratio of nuclear area to whole cell YAP/TAZ.
14. SER texture features (Spot, Hole, Edge, Ridge, Valley, Saddle, Bright, Dark) for α-tubulin and actin cytoplasm stains; and Hoechst, α-tubulin, and actin nucleus stains, at 0 and 1 pixel scale.
15. Haralick texture features (Correlation, Contrast, Variance, Homogeneity) for α-tubulin cytoplasm stain; and α-tubulin and actin nucleus stains, at 1 pixel scale.
16. Gabor texture features for cytoplasm actin stain.
17. Area of membrane region 1 and 2 in (px^2^).
18. Neighbour fraction.

#### Cell morphology features (types 1–4)

Five morphological properties for the nucleus, cytoplasm, and whole cell regions were calculated: area, roundness, width, length, and the ratio of width to length (15 features). Additionally, a membrane region mask was created to quantify protrusiveness, and its area was calculated. This membrane region mask was defined as a mask that extends slightly beyond the cytoplasm and encompasses a percentage of the cytoplasm. The inner border and outer borders were set at 10% and −5% relative to the distance from the cytoplasm edge to the centroid of the cell. Considering the inner border starts at the centroid of the cell and encompasses the whole cell when at 100%, the inner border was set at 10% of the distance from the cytoplasm edge to the centroid of the cell, and the outer border was set at −5%.

#### Cell region-specific features (types 5–13)

Feature calculations were made for YAP/TAZ, Hoechst and α-tubulin intensities for different cell regions. Specifically: Hoechst intensity was calculated for the nuclear region; α-tubulin intensity for the cytoplasm and whole cell regions (combined nuclear and cytoplasm regions); and YAP/TAZ intensity was calculated for the nuclear, cytoplasm, and whole cell regions. We identified two additional regions in which we calculated YAP/TAZ intensity: the ‘ring region’ and ‘eroded nuclear region’. The ring region was defined by creating a mask around the nuclear segment and calculating a percentage of the distance from the cytoplasm edge to the centroid of the cell (inner border: 40%, considering 100% to encompass the whole cell). The outer border of the ring region was determined as 20% of the distance from the cytoplasm edge to the inner border. The eroded nuclear region was established by eroding the nuclear region by 2%, such that the eroded nuclear region is a smaller version of the nuclear region and consequently is not in direct contact with the ring region. This was done to compensate for segmentation errors. However, for the purposes of our analyses there was no difference between using the eroded nuclear region or the nuclear region itself. The data for both is included so that users can choose their feature of preference.

To quantify the nuclear translocation of YAP/TAZ, ratios were taken for the nuclear to ring region, and eroded nuclear to ring region measurements. Using the ring region has been shown to improve nuclear translocation measurement accuracy^17^. This is due to the ring region having a similar thickness to the nucleus and thus being more comparable than using the whole cytoplasm versus the nucleus. The logarithm of these features was also calculated as a derivative feature. The log10 of the ratio of nuclear region YAP/TAZ intensity to ring region YAP/TAZ intensity is hereafter referred to as ‘YAP/TAZ ratio’. The ratio of YAP/TAZ intensity to nuclear and whole cell area were also calculated.

#### Texture features (types 14–16)

‘Texture features’ for both the nuclear and cytoplasmic regions were also measured. Three types of texture feature were calculated: Gaussian Derivative Textures, termed SER, (Saddle-Edge-Ridge) features in Columbus; Gabor features^25^; and Haralick features^26–28^. SER features are calculated as the mean region intensity following convolution with a Gaussian kernel (which identify spot and hole textures), and derivatives of this kernel (which identify saddle, edge, ridge, valley, bright and dark textures). These textures were calculated for α-tubulin, Hoechst and actin channels. Two smoothing scales were used, 0 pixels to identify smaller objects, and 1 pixel to identify larger objects. The Gabor minimum and maximum features are the mean, minimum, and maximum values of a wavelet kernel projected at 8 different angles; these features identify actin structures, and are only calculated for this channel^25^. SER and Gabor features were normalised to within the kernel used to calculate them, thus ensuring only local textures were detected. Haralick features are calculated from the co-occurrence matrix, which describes how similar neighbouring pixels are within an image. Four features were defined from this matrix: correlation, contrast, variance, and homogeneity^27,28^. These features were calculated for α-tubulin, and actin channels.

#### Microenvironment features (types 17–18)

To determine the fraction of a cell in contact with another cell, or the ‘neighbour fraction’^17^, two membrane region masks were generated. A membrane region 1 with an outer and inner border of 0 and 1 pixels respectively, and a membrane region 2 with an outer and inner border of −1 and 0 pixels respectively. The area of these membrane regions was obtained and the neighbour fraction was then calculated as 1 minus the ratio of the area of membrane region 1 over twice the area of membrane region 2.

#### Classification of cell shape

Identification of cell shapes within a population can aid in visualisation and interpretation of datasets^19,29^. Here the population of single cells was classified into five visually distinctive reference shapes for users of the dataset to utilise in data analysis, or compare to their own sub-population identification results, utilising the imaging data provided here. The five shapes identified were: 1) ‘spindly’ shape, elongated cells with typically two protrusions; 2) ‘large spread’ shape, cells with a large area which are often circular; 3) ‘triangular’ shape, cells with three distinct protrusions; 4) ‘fan’ shape, asymmetrically-shaped cells with nucleus to one side; 5) ‘small round’ shape, cells with low cell area and high roundness. To classify single cells into these reference shapes linear classifiers were trained on approximately 1,500 manually selected cells using Columbus software. The trained classifiers were then applied to all single cells within a given well of the screen. The total number of cells classified into each shape is then given as a feature for each well. Of note, Columbus software classifies each cell into a particular shape only once.

**Figure 2:**
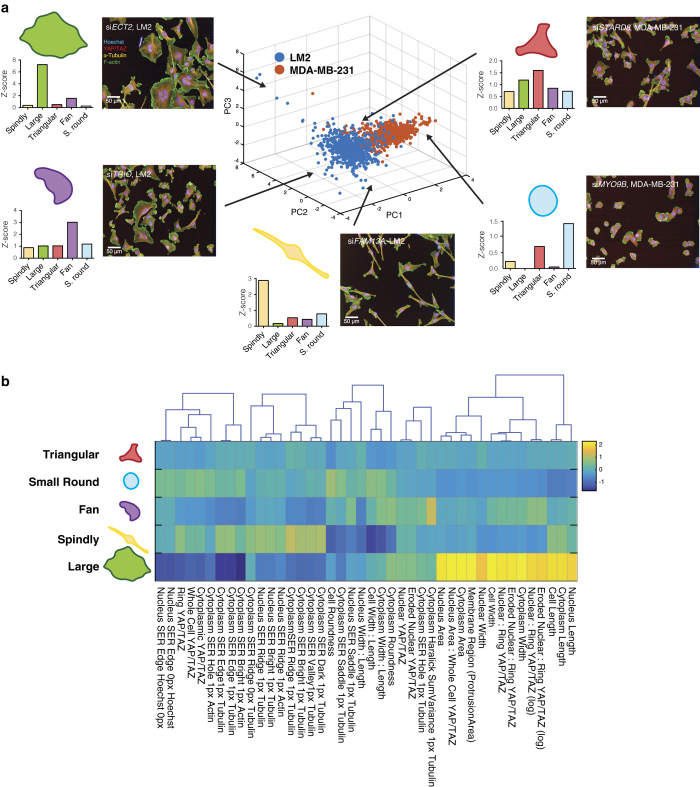
Linear classification of shapes. (**a**) PCA was applied to the Z-score of shapes that cells were classified into for ONTARGET*Plus* screens for LM2 (blue) and MDA-MB-231 (orange). siRNA treated populations enriched for each shape (as judged by Z-scores) are depicted: spindly in yellow, large in green, triangular in red, fan in purple, and small and round in blue. (**b**) Heat map and dendrogram of the features Columbus software used to classify cells into the chosen 5 shapes, based on the cluster means for negative and positive control data (wild-type and *ECT2* siRNA which enriches for the large spread morphology absent in wild type). For example, cells classified into large shape scored high for cytoplasm and cell length and width, and cytoplasm and nuclear area. On the other hand, cells classified into spindly shape scored low for cytoplasm and cell width to length ratio, and cytoplasm and cell roundness. Yellow and dark blue represent a high and a low score respectively in arbitrary units.

The proportion of cells within a given shape varies greatly between the two cell lines used, as can be seen from the Principal Component Analysis (PCA) depicted in Fig. 2a (LM2 in blue and MDA-MB-231 in orange). Within individual cell lines, siRNA depletion of genes leads to a high degree of variability. For example, *TRIO* depletion enriches for the fan shape, whilst depletion of *FAM13A* enriches for the spindly morphology (Fig. 2a). To further provide an understanding of the contribution of each feature to classification of cells into a shape, the cluster means for negative and positive control data (wild-type and *ECT2* siRNA which enriches for the large spread morphology absent in wild type), are shown in Fig. 2b.

#### Initial hit selection

As part of initial exploration of the data we identified siRNAs (‘hits’) that resulted in significantly different: (1) cell shapes; (2) YAP/TAZ ratio (YAP/TAZ activation); (3) and sum of nuclear region and ring region YAP/TAZ intensities normalised to nuclear area (hereafter referred to as ‘total YAP/TAZ’). The sum of nuclear region and ring region YAP/TAZ intensities were normalised to nuclear area so as to account for nuclear size. Raw data obtained from the analysis performed by Columbus software for each plate, was normalised to each plate. Normalised values were then grouped per screen and then Z-scores were calculated using the control well average and standard deviation for each screen. A threshold of minimum 1.5 was used for selecting hits. Tables 1 and 2 show the Z-scores and fold change for the top hit for each condition for each cell line for the ONTARGET*Plus* screens, and its corresponding Z-score in the siGENOME screens. This excludes the controls *LATS1* and *YAP* which were top hits for YAP/TAZ ratio, and total YAP/TAZ respectively. The top hits were chosen from the ONTARGET*Plus* screens because it is likely these screens are less-prone to off-target effects than siGENOME screens. Except for *TRIO*, *ARHGAP5*, *NET1* in LM2 and *ARHGEF18, MCF2,* and *PIK3R2* in MDA-MB-231, all top hits for the ONTARGET*Plus* screens were also hits in siGENOME. Of note, siRNAs did not result in a decrease in cell number compared to control, so there was no need to account for cell density when looking at YAP/TAZ intensities. Despite this, the YAP/TAZ cell density curves can still be used to see the linear relationship between YAP/TAZ localisation and cell density.

### Code availability

Matlab code for quality control available on: Github: https://github.com/samocooper/GEFGAP_testing_script.

## Data Records

Every Data Record contains one Excel spreadsheet where each replicate plate can be found on a different tab: 1A, 1B, 2A, and 2B. Headings are labelled as described under list of features extracted. The number of cells plated for the YAP/TAZ cell dilution curves on columns 1, 2, 23 and 24 can be found in the same column as gene names, instead of a gene name for those particular wells. Spreadsheets can be found under, and be downloaded from the ‘Attachments’ tab for each screen.

### Data record 1

Raw data for the siGENOME RhoGEF and RhoGAP RNAi screen on LM2 cells is available from the University of Dundee Image Data Repository (IDR) under http://dx.doi.org/10.17867/10000104A. (Data Citation 1). The spreadsheet is labelled LM2_siGENOME_features.txt.

### Data record 2

Raw data for the ONTARGET*Plus* RhoGEF and RhoGAP RNAi screen on LM2 cells is available from IDR under http://dx.doi.org/10.17867/10000104B (Data Citation 2). The spreadsheet is labelled LM2_ONTARGETPlus_features.txt.

### Data record 3

Raw data for the siGENOME RhoGEF and RhoGAP RNAi screen on MDA-MB-231 cells is available from IDR under http://dx.doi.org/10.17867/10000104C (Data Citation 3). The spreadsheet is labelled MDA-MB-231_siGENOME_features.txt.

### Data record 4

Raw data for the ONTARGET*Plus* RhoGEF and RhoGAP RNAi screen on MDA-MB-231 cells is available from IDR under http://dx.doi.org/10.17867/10000104D (Data Citation 4). The spreadsheet is labelled MDA-MB-231_ONTARGETPlus_features.txt.

### Data record 5

Sample images for genes whose depletion enriches for a particular shape, YAP/TAZ ratio, or total YAP/TAZ are available from Figshare under https://dx.doi.org/10.6084/m9.figshare.c.3305517 (Data Citation 5). The images for the top hits found in Tables 1 and 2 of this manuscript can be found in the sample images, alongside other genes that were also hits, and mock transfected cells. Each image is labelled with the name of the siRNA, the library (siGENOME or ONTARGET*Plus*), the cell line, and what they enrich for (shape, YAP/TAZ ratio, or total YAP/TAZ). Images from mock transfected cells for both LM2 and MDA-MB-231 are labelled as ‘Cells’ followed by the cell line. Each image corresponds to one field from one well for each particular gene. Where more than one field has been included for a particular gene, the name of the file is the same followed by a 2 in parenthesis. Images for genes that enrich for a particular cell shape or are mock transfected, depict an overlay of Hoechst, α-tubulin, F-actin, and YAP/TAZ. Images for YAP/TAZ ratio and total YAP/TAZ, depict YAP/TAZ stain only and were all taken with the same contrast parameters, such that they can all be compared. Images for depletion of *LATS1* and *YAP* can be found in the sample images as controls for high YAP/TAZ ratio and low total YAP/TAZ respectively. Images for genes whose depletion does not cause a change in YAP/TAZ ratio or total YAP/TAZ levels have also been included as visual aids.

## Technical Validation

### Replicate plate reproducibility

**Figure 3:**
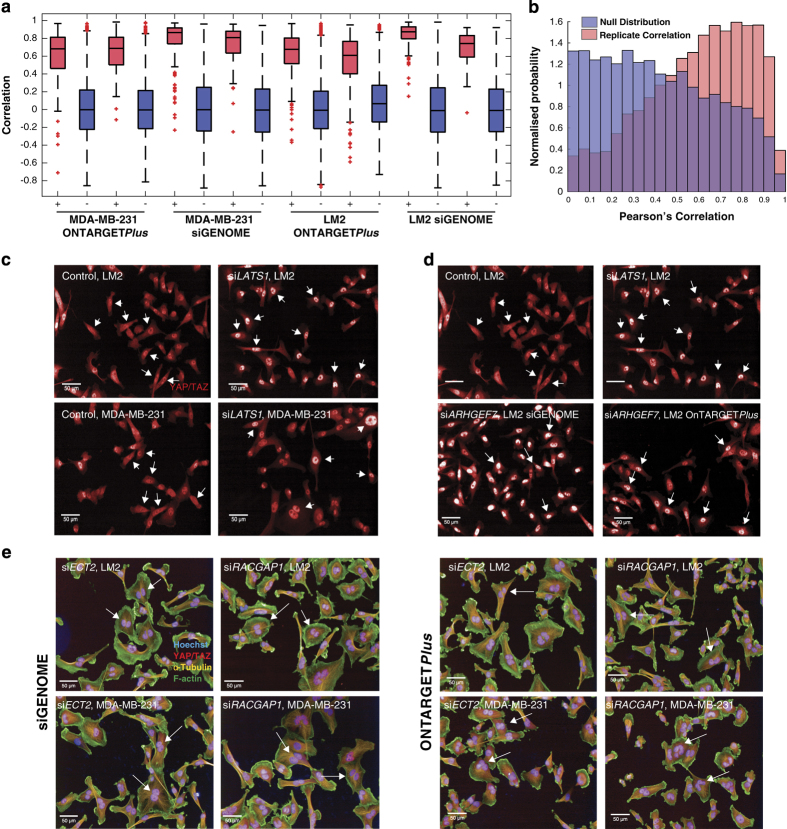
siRNA depletion is reproducible between replicate plates and over the screen. (**a**) Correlation matrix between Z-scores of well-averaged feature vectors between duplicate plates. The positive distribution (+) represents the correlation between replicate wells and the negative distribution (−) determines the null-value correlation. Pairwise *t*-tests for the correlation increases from null gave a *P* value less than 0.001. (**b**) Pearson correlations between technical replicates and randomly drawn siRNAs. The positive distribution has a median correlation of 0.594, and a strong negative skew of −1.02. The null distribution has a near zero median of 0.169 and no skew (0.02). *P*<0.001 (pairwise *t*-test) between the positive distribution and the null distribution. (**c**) Representative images of the effect of knocking down *LATS1* on YAP/TAZ nuclear translocation in LM2 and MDA-MB-231 cells compared to control cells (containing only transfection reagent). The intensity of YAP/TAZ stain in the nucleus is higher than for control cells (white arrows). (**d**) Representative images of YAP/TAZ translocation to the nucleus as a result of *ARHGEF7* knockdown in both the siGENOME and ONTARGET*Plus* screens in LM2 cells, compared to control cells and si*LATS1* transfected cells are included as a comparison. White arrows point to cells whose YAP/TAZ intensity is representative of the condition stated. (**e**) Representative images of multinuclear cells (white arrows) as a result of *ECT2* and *RACGAP1* knockdown for LM2 and MDA-MB-231 in both the siGENOME and ONTARGET*Plus* screens.

Replicate plate reproducibility was calculated for each set of duplicate plates per screen, using the Pearson Correlation Coefficient. Specifically, well-averaged feature vectors were Z-score normalised to each plate. Subsequently, the correlation between well-averaged feature vectors was calculated between the duplicate plates. In the resulting correlation matrix, where each row is a well in plate A, and each column a well in plate B, the diagonal represents the correlation between replicate wells, whilst the off-diagonal is used to determine the null-value correlation. The positive and negative distribution of these values for each plate is presented in Fig. 3a. Pairwise *t*-tests showed all correlation increases from null were highly significant (*P*<0.001). Furthermore in no cases did the interquartile ranges of positive to null distributions overlap, indicating a high level of reproducibility.

### siRNA reproducibility

To test the reproducibility of siRNA effect, the distribution of Pearson correlations between technical replicates, and randomly drawn siRNAs were compared. For the positive distribution, correlations of well-averaged feature vectors were compared for technical siRNA replicates across the screen. For the null distribution, feature vectors were randomised, and the same calculation was performed. The positive distribution showed a highly significant increase compared to the null distribution (pairwise *t*-test, *P*<0.001). For the positive distribution, a median correlation of 0.594 was observed, and a strong negative skew (−1.02) was also present; suggesting high levels of correlation were observed between the majority of replicates (Fig. 3b). Meanwhile, the null distribution showed a median near zero (0.169) and no skew (0.02), indicating siRNA treatments induced reproducible changes in cell morphology.

### Control performance

The performance of negative and positive controls was assessed by calculating the Z’ factor, a widely used test to determine the dynamic range of RNAi screens^30,31^. The Z’ factor is a statistical measure which requires the ratio of the sum of the standard deviations between controls and the difference between the means to be at least three standard deviations from each other^30^. Values above 0.3 are deemed acceptable, with values between 0.5 and 1.0 being desirable^31^. The average of the values for each control are shown in Tables 3 and 4 for the MDA-MB-231 and LM2 cell lines respectively. All plates performed well for at least one positive versus negative control for each cell line, with ONTARGET*Plus* plates performing better than siGENOME overall. si*YAP* performance was assessed using the total YAP/TAZ; while si*LATS1* performance was assessed using the YAP/TAZ ratio. si*YAP* and si*LATS1* were not included in the LM2 siGENOME screen, so si*ECT2* was used to calculate the Z’ factor instead. si*ECT2* performance was assessed based on multinuclearity. LM2 screens performed very well for si*ECT2* versus mock, with an average of 0.71 Z’ factor for siGENOME, and 0.64 for ONTARGET*Plus*. Given that the Z’ factor for both LM2 screens was above 0.50 for si*ECT2*, it is reasonable to expect that if si*YAP* and si*LATS1* had been included in the siGENOME screens, the Z’ factors would have been similar to that in the ONTARGET*Plus* screens.

Our ability to detect YAP/TAZ by immunolabelling was assessed first by calculating Z-scores of total YAP/TAZ. Cells containing si*YAP* were hits for low total YAP/TAZ for both cell lines. Further, Z-scores of the YAP/TAZ ratio were also calculated. In this case, cells containing si*LATS1* were top hits for a high value of YAP/TAZ ratio. This was as expected, as when present LATS1 sequesters YAP/TAZ in the cytoplasm^15^. Further, the change in YAP/TAZ localisation can clearly be observed by looking at the images (Fig. 3c).

Validating our ability to detect changes in YAP/TAZ localisation is that depletion of *ARHGEF7* is a hit for high YAP/TAZ ratio, for both the siGENOME and ONTARGET*Plus* screens in the LM2 cell line (Fig. 3d). Knockdown of *ARHGEF7* has previously been reported to increase nuclear YAP/TAZ^32^.

### siRNA gene knockdown efficiency

To determine siRNA knockdown efficiency, analyses were run to determine multinucleate cell percentage. The cytokinesis regulators *ECT2* and *RACGAP1* (ref. 33) were top hits for both cell lines for multinucleate cell percentage Z-scores. The high number of multinucleate cells for these siRNAs was also observed by looking at the images (Fig. 3e).

As an additional form of validating the reliability of the screens, mRNA levels were measured following quantitative knockdown of *ECT2, DOCK5*, and *ARHGEF7* using ONTARGET*Plus* siRNAs (Supplementary Fig. 1). *ECT2* was chosen based on the fact that we use this as a control for transfection efficiency and it consistently being a hit for the large spread shape phenotype. *DOCK5* is top hit for spindly shape in MDA-MB-231s. *ARHGEF7* was chosen because it has been reported to increase YAP/TAZ^32^, which is further supported by the screens presented here. Knockdown efficiency of all siRNAs ranged between 88 and 99% for both cell lines, indicating that siRNA gene knockdown was efficient and the screens reliable. As a way of providing evidence of siRNA specificity, quantitative knockdown of individual siRNAs targeting *ARHGEF7* (deconvoluted ONTARGET*Plus* SMARTpool) was performed (Supplementary Fig. 1c). All individual siRNAs targeting *ARHGEF7* resulted in efficient gene knockdowns. We have previously observed that depletion of *ARHGEF7* using all individual siRNAs of the ONTARGET*Plus* SMARTpool, results in a consistent phenotype^34^ supporting the idea that the effect of *ARHGEF7* depletion on YAP/TAZ levels is on-target. Meaning, it is unlikely that all single siRNAs from the pool effectively deplete *ARHGEF7*, but have a similar off-target effects.

## Usage Notes

The raw RNAi screening data (Data records 1–4) are provided for users to be able to apply their own normalisation strategies and thresholds for altered YAP/TAZ localisation, cell shape and texture features. Binning each population into a limited number of shapes is a supervised data reduction strategy which we have used in the past, and have found provides biologically meaningful separation of the data^18,19^. However, other data reduction methods, especially unsupervised methods, may also be appropriate means to reduce data.

Users should be aware that although the data for the whole 384 well plates is provided, not all negative control wells are used for analysis. In particular, it is customary to exclude the two edge rows around the plate. Further, plates numbered 2A and 2B contain less siRNAs than 1A and 1B and consequently contain more negative controls. The negative control wells, mock treated with transfection reagent but no siRNA, used for analysis can be found within columns 3 to 22, and rows C to N in plates 1A and 1B of the siGENOME screens; within columns 3 to 22 and rows C to M in plates 2A and 2B of both siGENOME and ONTARGET*Plus* screens; and within columns 3 to 22 of rows B and O for plates 1A and 1B of the ONTARGET*Plus* screens.

## Additional Information

**How to cite this article:** Pascual-Vargas, P. *et al.* RNAi screens for Rho GTPase regulators of cell shape and YAP/TAZ localisation in triple negative breast cancer. *Sci. Data* 4:170018 doi: 10.1038/sdata.2017.18 (2017).

**Publisher’s note:** Springer Nature remains neutral with regard to jurisdictional claims in published maps and institutional affiliations.

## Supplementary Material



Supplementary Figure 1

## Figures and Tables

**Table 1:** Summary of top hits for each shape, YAP/TAZ ratio, and total YAP/TAZ for LM2 ONTARGET*Plus* and siGENOME screens.

	**Gene Name**	**Gene ID**	**LM2 ONTARGET*****Plus***		**LM2 siGENOME**	
			**Fold Change**	**Z-score**	**Fold Change**	**Z-score**
Spindly	*FAM13A*	10144	3.13	10.6	2.61	6.97*
Large, spread	*ECT2*	1894	7.25	16.0	7.66	11.6*
Triangular	*TRIO*	7204	1.46	3.35	1.65	2.66*
Fan	*TRIO*	7204	2.58	3.29	1.27	1.13
Small, round	*ARHGAP5*	394	2.35	8.03	1.12	0.51
*YAP/TAZ ratio*						
High	*ARHGEF15*	22899	1.12	3.73	1.12	2.17*
Low	*ARHGAP33 /SNX26*	115703	0.48	−5.65	0.86	−2.94*
*Total YAP/TAZ*						
High	*NET1*	10276	1.40	3.45	1.19	0.99
Low	*MCF2*	4168	0.38	−7.85	0.79	−2.38*
Top hits were chosen from the ONTARGET*Plus* library. Hits considered to be common across siRNA libraries are denoted by an asterisk.						

**Table 2:** Summary of top hits for each shape, YAP/TAZ ratio, total YAP/TAZ for MDA-MB-231 ONTARGET*Plus* and siGENOME screens.

	**Gene Name**	**GeneID**	**MDA-MB-231 ONTARGET*****Plus***		**MDA-MB-231 siGENOME**	
			**Fold Change**	**Z-score**	**Fold Change**	**Z-score**
Spindly	*DOCK5*	80005	2.78	8.70	1.62	2.57*
Large, spread	*ECT2*	1894	5.47	7.83	8.64	10.7*
Triangular	*MCF2*	4168	1.54	5.14	0.97	0.40
Fan	*ECT2*	1894	3.15	6.98	3.02	7.58*
Small, round	*ARHGEF18*	23370	1.57	4.01	0.48	−4.45
*YAP/TAZ ratio*						
High	*PIK3R2*	5296	1.67	5.67	0.85	−1.34
Low	*MYO9B*	4650	0.62	−3.39	0.73	−2.84*
*Total YAP/TAZ*						
High	*FARP1*	10160	1.31	1.93	1.60	4.19*
Low	*PIK3R2*	5296	0.61	−5.46	0.74	−3.41*
Top hits were chosen from the ONTARGET*Plus* library. Hits considered to be common across siRNA libraries are denoted by an asterisk.						

**Table 3:** Summary of average Z’ factor to mock for MDA-MB-231 siGENOME and ONTARGETPlus screens.

**Average Z’ factors for MDA-MB-231 screens**			
**siGENOME**		**ONTARGET*****Plus***	
***YAP*****/mock**	***LATS1*****/mock**	***YAP*****/mock**	***LATS1*****/mock**
0.53	0.29	0.76	0.57

**Table 4:** Summary of average Z’ factor to mock for LM2 siGENOME and ONTARGET*Plus* screens.

	**Average Z’ factors for LM2 screens**		
**siGENOME**	**ONTARGET*****Plus***		
***ECT2*****/mock**	***ECT2*****/mock**	***YAP*****/mock**	***LATS1*****/mock**
0.71	0.64	0.67	0.40
