# Supplementary material for: RNAi screens for Rho GTPase regulators of cell shape and YAP/TAZ localisation in triple negative breast cancer
Source: Sci Data. 2017 Mar 1;4:170018. doi: 10.1038/sdata.2017.18 (PMC5332010; doi:10.1038/sdata.2017.18)
Supplement: Supplementary Figure 1 [file sdata201718-s2.pdf]

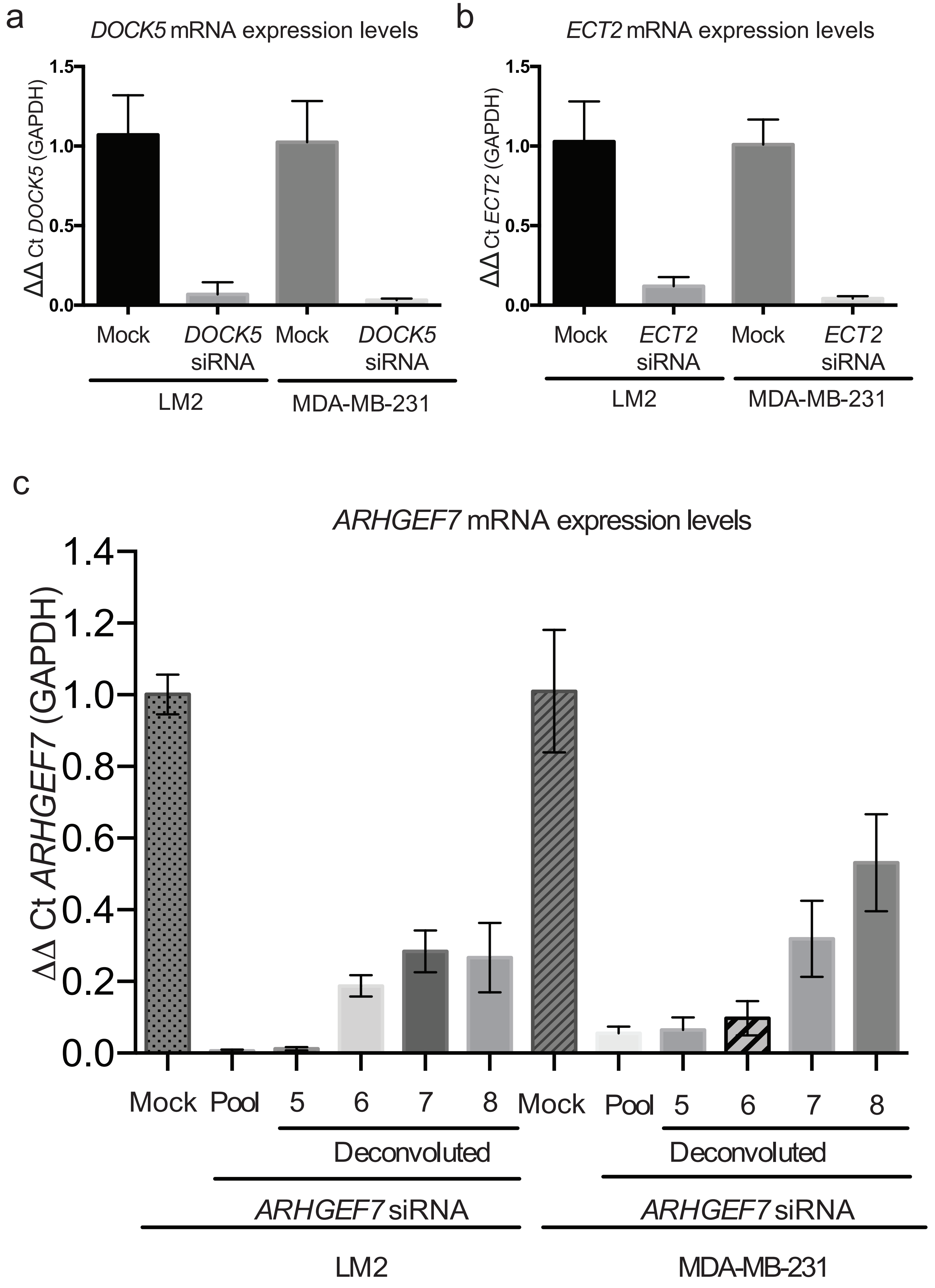

a
DOCK5 mRNA expression levels
ΔΔ Ct DOCK5 (GAPDH)
1.5
1.0
0.5
0.0
Mock
DOCK5 siRNA
Mock
DOCK5 siRNA
LM2
MDA-MB-231
b
ECT2 mRNA expression levels
ΔΔ Ct ECT2 (GAPDH)
1.5
1.0
0.5
0.0
Mock
ECT2 siRNA
Mock
ECT2 siRNA
LM2
MDA-MB-231
c
ARHGEF7 mRNA expression levels
ΔΔ Ct ARHGEF7 (GAPDH)
1.4
1.2
1.0
0.8
0.6
0.4
0.2
0.0
Mock
Pool
5
6
7
8
Mock
Pool
5
6
7
8
Deconvoluted
Deconvoluted
ARHGEF7 siRNA
ARHGEF7 siRNA
LM2
MDA-MB-231

Supplementary Figure 1: siRNA validation. (a) *DOCK5* mRNA expression levels normalised to *GAPDH* for mock-transfected and *DOCK5* siRNA containing LM2 and MDA-MB-231s. (b) *ECT2* mRNA expression levels normalised to *GAPDH* for mock-transfected and *ECT2* siRNA containing LM2 and MDA-MB-231s. (c) *ARHGEF7* mRNA expression levels normalised to *GAPDH* for LM2 and MDA-MB-231s that were mock-transfected, contained *ARHGEF7* siRNA, and individual siRNAs targeting *ARHGEF7*. Data are shown as means ± standard deviation from at least 3 technical replicates.
